# Design of Quasi-Zero-Stiffness Metamaterials Featuring Adjustable Thermal Expansion

**DOI:** 10.3390/ma19081613

**Published:** 2026-04-17

**Authors:** Ziqi Li, Lu Zhang, Zheng He, Haitao Wang, Zhaotuan Ding, Hongtao Wang, Yongmao Pei

**Affiliations:** 1 Institute of Nuclear and New Energy Technology, Tsinghua University, Beijing 100084, China; 2 School of Mechanics and Engineering Science, Peking University, Beijing 100871, China; 3 Chinergy Co., Ltd., Beijing 100193, China

**Keywords:** QZS, ZTE, cylindrical metamaterial, stable vibration isolation performance

## Abstract

To address the limitations of conventional metamaterials in thermo-mechanical coupling environments, this study proposes a multifunctional metamaterial structure through material selection and structural optimization, demonstrating stable vibration isolation performance under thermal fluctuations. The thermal deformation mechanisms and zero thermal expansion (ZTE) behavior of curved-beam unit cell are systematically examined through the chained beam constraint model (CBCM). A novel dual-zero metamaterial featuring both quasi-zero-stiffness (QZS) and ZTE characteristics is developed using curved-beam unit cell design. A parametric analysis, through finite element modeling, systematically investigated the effects of geometric parameters and material properties on the thermal expansion deformation and mechanical responses in the curved-beam unit cell structure. Furthermore, cylindrical metamaterials featuring dual-zero properties were engineered, and their deformation control mechanisms and vibration characteristic evolution across a broad temperature range were systematically studied. The simulation results indicate that while the Al–Al structure exhibits a significant resonance peak shift of up to 64.32% at 200 °C, the Al–Steel zero-stiffness design restricts this shift to only 7.72%. Furthermore, the Steel–Invar configuration demonstrates exceptional vibrational stability, with its center frequency shifting marginally from 5.58 Hz to 5.61 Hz at 200 °C. This methodology presents a viable solution for engineering metamaterials in extreme-temperature environments.

## 1. Introduction

Maintaining the vibration isolation property stability of structures in thermal environment is of critical importance in precision instruments applications [1,2,3,4]. The vibration isolator located between the support base and the upper precision instruments must maintain stable vibration isolation performance despite significant temperature fluctuations [5]. External thermal excitation induces alterations in both material properties and geometric configuration of structures, resulting in progressive degradation of vibration isolation performance that may culminate in catastrophic system failure. Therefore, designing a structure capable of ensuring both load-bearing capacity and stable low-frequency vibration isolation at elevated temperatures remains a considerable challenge [6].

QZS metamaterials have attracted substantial research interest due to the exceptional low-frequency vibration isolation performance and structural designability [7,8]. At present, most QZS structures usually achieve near-zero-stiffness characteristics by employing pre-buckled curved beams with tunable stiffness, where buckling or bending deformation dominates the nonlinear mechanical response [9]. Researchers systematically analyzed the stiffness platform and vibration isolation frequency range of the structure based on pre-buckling Euler beam unit cells [10,11]. By optimizing the geometric parameters of the curved beams, the intrinsic mechanism of their stiffness softening behavior was further elucidated [12,13]. Experimental verification confirmed that such structures exhibit outstanding vibration isolation performance, even in low and ultra-low-frequency ranges. To further advance design flexibility, a computational design framework [14] was proposed based on spline curve optimization, enabling the development of task-specific QZS metamaterials. Through programmable deformation control of support beams, this approach achieves an extended quasi-static stiffness platform with enhanced load capacity and improved low-frequency isolation performance [15].

However, the temperature sensitivity of QZS metamaterials remains underexplored in existing literature. Under extreme thermal gradients, curved beam within the unit cell structure may undergo thermally induced huge deformation which can cause contact or even damage to precision instruments or equipment installed in confined spaces and stiffness drift, critically degrading vibration isolation efficacy [16,17,18]. The investigation of NiTi alloy-based metamaterials [19] demonstrates that structural stiffness exhibits a positive temperature dependence, with increasing temperature resulting in elevated stiffness. This thermo-mechanical coupling effect induces a corresponding shift in the metamaterial’s resonant frequency, thereby compromising its vibration isolation performance. In parallel, Li [20] proposed a temperature-controlled QZS metamaterial beam and showed that thermal stimuli can be used to achieve broad-range tuning of low-frequency band gaps. Notably, metamaterials composed of low thermal expansion metals can satisfy the dual requirements of minimal thermal deformation and vibration isolation; they are limited by a narrow range of material choices and difficulties in modulating the thermal expansion coefficient [21].

To address temperature-induced structural deformation and vibration frequency shifts, researchers have developed composite material lattices with multi-material configurations [22,23]. A theoretical framework [24] was established through the “Miller Triangle” concept, which leverages thermal expansion coefficient mismatches between constituent materials to minimize equivalent thermal deformation, thereby providing systematic guidance for designing structures with tunable thermal expansion properties. Building upon this foundation, systematic investigations on zero-expansion metamaterials [25,26] were conducted. Their research established a fixed-support theoretical model for zero-expansion metamaterials. Building upon this foundation, they developed both two-dimensional and three-dimensional lattice structures with programmable thermal expansion properties and further proposed a cylindrical metamaterial exhibiting near-ZTE characteristics. Yu [27] integrated ZTE and vibration suppression functionalities into a single metamaterial, enabling the structure to maintain a stable vibration suppression effect under temperature changes. However, this design demonstrates effective vibration suppression only above 1000 Hz, failing to address low-frequency isolation requirements prevalent in instrumentation applications. Table 1 compares representative recent studies in the fields of QZS and thermally stable metamaterials. Thus, the critical challenge lies in synergistically combining thermal expansion control with low-frequency vibration isolation, which requires innovative design to bridge these functionally divergent objectives.

Building upon the principles of thermal expansion regulation and the load-bearing deformation mechanism of curved beams, this study proposes an innovative metamaterial design methodology that simultaneously achieves ZTE and QZS properties. For this purpose, the ZTE design approach is incorporated into the curved-beam QZS metamaterial unit cell, where the curved beam and supporting structure are designed with different material properties. Section 2 systematically investigates the effects of geometric parameters on the equivalent thermal expansion coefficient and zero-stiffness performance. Finite element simulations demonstrate that, by optimizing the ratio between curved-beam configuration and thermal expansion coefficient, the metamaterial achieves ZTE while maintaining stable vibration isolation performance under varying temperatures. In Section 3, the metamaterial unit cell is assembled into a cylindrical configuration with ZTE and QZS characteristics, followed by finite element analysis to validate the stable vibration isolation performance.

## 2. Materials and Methods

**Figure 1:**
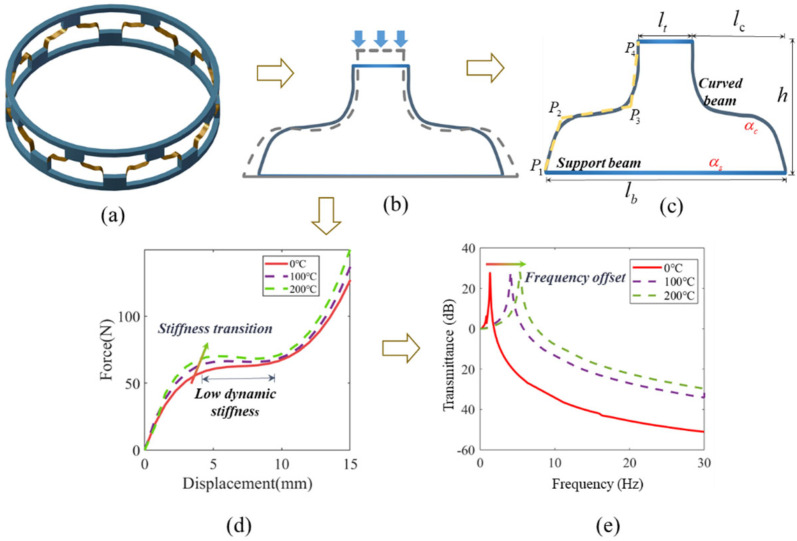
(**a**) Cylindrical curved-beam metamaterial structure. (**b**) The structure deforms under the coupled action of temperature and load. (**c**) Essential parameters of metamaterial parameters and curved-beam control nodes. (**d**) The load–displacement curve changes with temperature. (**e**) The offset of transmissibility.

As shown in Figure 1a,b, the metamaterial cylindrical shell structure is composed of multiple curved-beam unit cells connected in parallel. The unique spline curve design of the curved beams endows the structure with high static stiffness and low dynamic stiffness characteristics, while the upper and lower horizontal beam ($l_{t}$ and $l_{b}$) supports provide a load-bearing platform and fixed constraints (Figure 1c). The functional properties of the spline curved-beam is controlled by four key points at their ends and midspan, with their overall geometry characterized by parameters $l_{c}$ and h. As illustrated in Figure 1d, under thermal loading, the relative expansion-induced deformation alters the structural stiffness and preload distribution, leading to a transition in the force–displacement response. Specifically, the QZS platform of the curved-beam unit cell changes from a positive stiffness regime to a negative stiffness regime, accompanied by a corresponding shift in resonant frequency (Figure 1e). To simultaneously achieve both QZS and ZTE properties in the curved-beam unit cell structure, a novel dual-material curved-beam unit cell design has been developed based on the thermal expansion triangle principle for ZTE structures. Through strategic optimization of structural parameters and material properties, a deliberate thermal expansion mismatch between the supporting beams and curved beams is introduced. This engineered mismatch effectively compensates for vertical thermal deformation, enabling the concurrent preservation of both the zero-stiffness platform and stable vibration isolation performance under thermal fluctuations.

**Figure 2:**
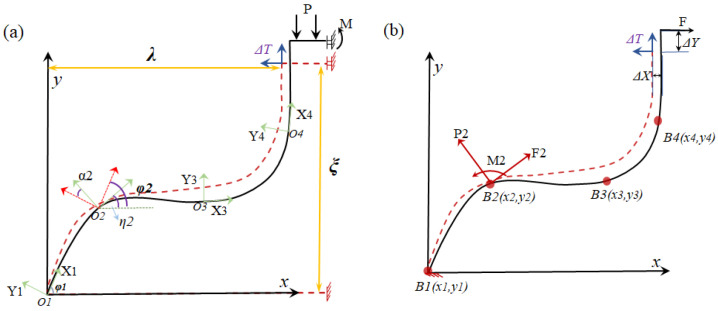
(**a**) Structural parameters and deformation diagram of discretized spline curved beam; (**b**) equivalent load diagram for discretized spline curved beam.

Figure 2 presents the deformation and equivalent load analysis of the discretized spline curved-beam unit cell. The uniform temperature field is applied throughout the beam element, with deformation analysis restricted to axial displacements in the local coordinate system. In the global coordinate system, transverse and vertical displacements occur at the ends of the curved beam. As shown in Figure 2a, the spline curved beam is discretized into multiple uniform beam elements. The initial angle of the ith beam element is $\eta_{i}$. Upon load application, the change in the curve beam angle is $\alpha_{i}$, resulting in a total curve angle relative to the global coordinate system of $\varphi_{i}$. Owing to the symmetry of the curved beam cell, its upper support structure constitutes a sliding fixed support, while the lower support is a fixed support. As illustrated in Figure 2b, to investigate the influence of the upper and lower support structures on the deformation of the curved beam under temperature fluctuations within a simplified model, the constraint-induced thermal stress from the supports is equivalently modeled as a horizontal load F applied to the upper end of the curved beam, and a fixed support boundary condition is applied at the bottom end of the curved beam.

For calculating large flexible deformations of curved beams, the CBCM [28,29,30] is introduced to solve the force–displacement relationship. During the solution process, temperature-induced normalized strain is incorporated into the axial displacement. For the ith element, the transverse force, the axial force, and the end moment forces are $p_{i}$, $f_{i}$, and $m_{i}$, with axial and transverse deflections, and the end angles are $x_{i}$, $y_{i}$, and $\alpha_{i}$ in the local coordinate frame. Its normalized external forces can be expressed as:

(1) $$p_{i}=\frac{P_{i}L^{2}}{N^{2}EI},f_{i}=\frac{F_{i}L^{2}}{N^{2}EI},m_{i}=\frac{M_{i}L^{2}}{NEI},x_{i}=\frac{N\Delta X_{i}}{L},y_{i}=\frac{N\Delta Y_{i}}{L},\alpha_{i}=\alpha_{i}$$

where L is the length of spline curve, N represents the number of the curve, I is the moment of inertia of the section, E denotes the material modulus, for which is assumed that its influence on temperature can be neglected.

For the overall structure, it can be expressed as:

(2) $$p_{0}=\frac{PL^{2}}{N^{2}EI},f_{0}=\frac{FL^{2}}{N^{2}EI},m_{0}=\frac{ML^{2}}{NEI},x_{0}=\frac{N\lambda }{L},y_{0}=\frac{N\xi }{L},\theta_{0}=\theta_{0}$$

where λ is the length in the x-direction of the global coordinate system, ξ is the length in the y-direction of the global coordinate system, and $\theta_{0}=\sum_{i=1}^{N}\alpha_{i}$ is the cumulative angle.

The CBCM equation of the curved beam is:

(3) $$\begin{array}{l} \left[\begin{array}{c} f_{i}\\ m_{i} \end{array}\right]=\left[\begin{array}{cc} 12 & -6\\ -6 & 4 \end{array}\right]\left[\begin{array}{c} y_{i}\\ \alpha_{i} \end{array}\right]+\frac{p_{i}}{30}\left[\begin{array}{cc} 36 & -3\\ -3 & 4 \end{array}\right]\left[\begin{array}{c} y_{i}\\ \alpha_{i} \end{array}\right]+\frac{p_{i}^{2}}{6300}\left[\begin{array}{cc} -9 & 4.5\\ 4.5 & -11 \end{array}\right]\left[\begin{array}{c} y_{i}\\ \alpha_{i} \end{array}\right]\\ x_{i}=\frac{s^{2}p_{i}}{12}-\frac{1}{60}\left[y_{i}\;\alpha_{i}\right]\left[\begin{array}{cc} 36 & -3\\ -3 & 4 \end{array}\right]\left[\begin{array}{c} y_{i}\\ \alpha_{i} \end{array}\right]-\frac{p_{i}}{6300}\left[y_{i}\;\alpha_{i}\right]\left[\begin{array}{cc} -9 & 4.5\\ 4.5 & -11 \end{array}\right]\left[\begin{array}{c} y_{i}\\ \alpha_{i} \end{array}\right]+\alpha_{c}\Delta T \end{array}$$

where s=NS/L, S is the thickness of curved beam, $\alpha_{c}$ is the coefficient of thermal expansion of the curved beam, ΔT is the temperature gradient.

The static equilibrium conditions at the fixed support boundary conditions of the spline curve are:

(4) $$p_{1}=p_{o},f_{1}=f_{o},m_{N}=m_{o}$$

The control equation of geometric constraints can be written as:

(5) $$\left\{\begin{array}{c} \sum_{i=1}^{N}\left[(1+u_{xi}+\alpha_{c}\Delta T)L_{i}\cos\varphi_{i}-(u_{yi})L_{i}\sin\varphi_{i}\right]=\lambda \\ \sum_{i=1}^{N}\left[(1+u_{xi}+\alpha_{c}\Delta T)L_{i}\sin\varphi_{i}+(u_{yi})L_{i}\cos\varphi_{i}\right]=\xi \\ \sum_{i=1}^{N}\alpha_{i}=\theta_{0} \end{array}\right.$$

where $\theta_{1}=0,\theta_{i}=\sum_{k=1}^{i-1}\alpha_{k}(i=2,3,\ldots ,N)$, $\varphi_{i}=\theta_{i}+\eta_{i}$.

The force and moment balance requirements for the ith element and the 1st element are:

(6) $$\left[\begin{array}{ccc} \cos\varphi_{i} & \sin\varphi_{i} & 0\\ -\sin\varphi_{i} & \cos\varphi_{i} & 0\\ \left(1+x_{i}+\alpha_{c}\Delta T\right) & -(y_{i}) & 1 \end{array}\right]\left[\begin{array}{c} f_{i}\\ p_{i}\\ m_{i} \end{array}\right]=\left[\begin{array}{c} f_{1}\\ p_{1}\\ m_{i-1} \end{array}\right]$$

This model comprises *N* beam elements and *N* + 1 nodes, with known external loads applied. At each node, six unknown quantities exist: (p,f,m,x,y,θ); the system thus contains 6*N* + 6 unknowns. The system formulation yields a total of 6*N* governing equations, comprising 3*N* constitutive equations (Equation (3)) and 3*N* equilibrium equations (Equation (6)) for each element. In conjunction with the six prescribed boundary conditions, this complete set of 6*N* + 6 equations uniquely determines the complete mechanical state at each node.

**Figure 3:**
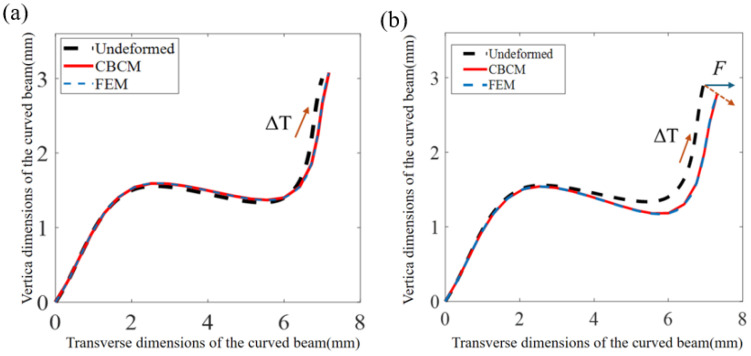
(**a**) Effect of temperature on the deformation of the spline curved beam. (**b**) Effect of temperature and equivalent support beam load on the deformation of the curved beam.

Figure 3a illustrates the effect of temperature on the deformation of the spline curved beam. The thermal expansion of the curved beam leads to an increase in both transverse and longitudinal displacements at its ends. The CBCM results show excellent agreement with FEM calculations, with an error of less than 1%. Figure 3b demonstrates that under the transverse equivalent load from the supporting beam, the spline curved beam undergoes transverse deformation while suppressing vertical deformation. Consequently, by adjusting thermal stress, zero thermal expansion in the vertical direction of the spline curved beam can be achieved.

**Figure 4:**
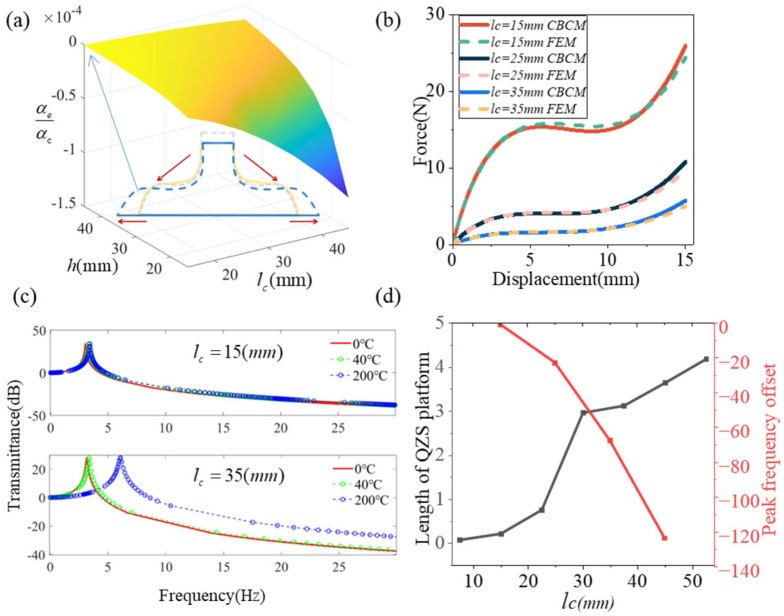
(**a**) Variation in vertical thermal expansion coefficients with geometric parameters at fixed thermal expansion coefficient ratio ($\alpha_{s}/\alpha_{c}=10$). (**b**) Load–displacement curves for specimens with different $l_{c}$. (**c**) The offset of resonance peaks under different $l_{c}$ lengths. (**d**) Variation in zero-stiffness platform length and peak frequency offset with different $l_{c}$.

Figure 4 systematically investigates the variation trends of the equivalent thermal expansion coefficient ($\alpha_{e}$) and force–displacement curves for the curved-beam unit cell, revealing that the thermal expansion coefficients ($\alpha_{s}=1.2\;\times \;10^{-5}/{}^{\circ}C,\alpha_{c}=1.2\;\times \;10^{-6}/{}^{\circ}C$ in Figure 4a) fundamentally determine the cell’s thermal expansion behavior. As shown in Figure 4b, the load-bearing capacity decreases significantly, as evidenced by the reduction in reaction force at the same displacement with increasing $l_{c}$. Meanwhile, the QZS platform becomes longer. This indicates that the larger $l_{c}$ weakens the overall structural stiffness and load-bearing capacity, but is beneficial for extending the QZS region. In addition, the comparison shows that the CBCM results are in good agreement with the FEM results under different parameter conditions, with consistent overall trends. The relatively larger discrepancies mainly appear in the large-displacement stage, but remain within 10%, indicating that the CBCM can accurately predict the nonlinear mechanical response of the structure. These local deviations slightly affect the interpretation of the overall variation pattern and performance evolution trend.

Figure 4c illustrates the effect of temperature on the peak frequency of the curved-beam metamaterial. To isolate the influence of thermally induced expansion from that of temperature-dependent material-property variations, the effects of temperature on the material modulus and other related parameters were neglected in the model, and only the structural thermal expansion effect was considered. When the beam length is relatively short ($l_{c}=15\mathrm{mm}$), the effect of temperature on the deformation of the curved beam is limited, and the overall structural stiffness remains nearly unchanged. Therefore, under the same load, the shift in the peak frequency is relatively small. In contrast, when the beam becomes longer ($l_{c}=35\mathrm{mm}$), the thermal deformation is transformed into the overall vertical deformation under the horizontal constraint, which leads to a change in the equivalent stiffness of the curved beam, and, consequently, the significant increase in the peak frequency shift.

Figure 4d illustrates the influence of $l_{c}$ on the length of the QZS platform ($\left|dF/du\right|<k_{0}$) and the resonance peak frequency offset ($\left(f_{0^{\circ }}-f_{200^{\circ }}\right)/f_{0^{\circ }}$). Since there is currently no clearly established criterion, $k_{0}=0.05\;N/m$ is assumed in the present study and used as the threshold for identifying the QZS platform. As $l_{c}$ increases, the length of the QZS platform generally shows an increasing trend. When $l_{c}$ is relatively small, the platform length grows slowly; when $l_{c}$ increases to approximately 25–30 mm, the platform length increases significantly. In contrast, the resonance peak frequency offset decreases continuously with increasing $l_{c}$, and the magnitude of the decrease becomes more pronounced. This indicates that the larger $l_{c}$ increases the thermal sensitivity of the structure, resulting in more severe frequency drift. This suggests a clear trade-off between the width of the QZS platform and thermal stability, and a balance between these two aspects is required in structural design.

**Figure 5:**
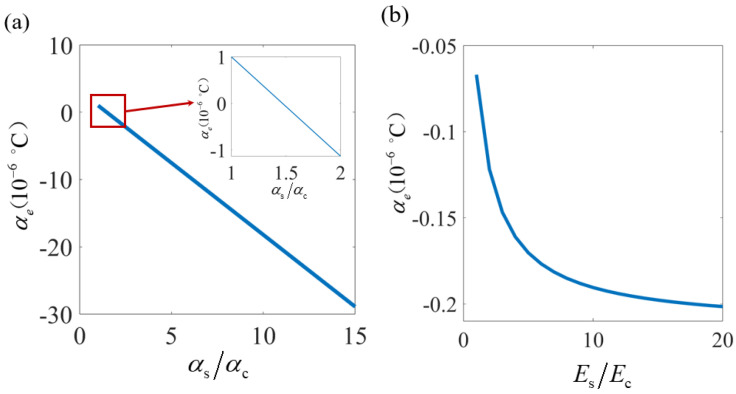
(**a**) Influence of thermal expansion coefficient ratio on the equivalent thermal expansion coefficient of curved-beam unit cell. (**b**) Modulation characteristics of elastic modulus ratio on the equivalent thermal expansion coefficient of curved-beam unit cell.

Considering that the curved beam should possess relatively high load-bearing capacity and a sufficiently wide stiffness platform, while also requiring a small peak frequency shift to facilitate subsequent design adjustment, the curved-beam unit cell with $l_{c}=20\mathrm{mm}$ and h=15mm was selected for further analysis. Figure 5a systematically investigates the coupled effects of thermal expansion coefficient ratio and material properties on the equivalent thermal expansion characteristics of the curved-beam unit cell. The results reveal a distinct transition from PTE to NTE in the vertical direction as the thermal expansion coefficient ratio increases. Notably, while the PTE behavior reaches a saturation limit of $1\times 10^{-6}/{}^{\circ}C$, the NTE regime demonstrates significantly enhanced tunability ($4\times 10^{-6}/{}^{\circ}C$), with its magnitude progressively amplifying at higher ratios.

Figure 5b reveals that the elastic modulus ratio nonlinearly modulates the thermal expansion behavior of the curved-beam unit cell. While this ratio does not alter the fundamental PTE/NTE characteristics, the NTE magnitude becomes progressively more pronounced with increasing modulus ratios. Notably, the thermal expansion behavior converges when $E_{s}/E_{c}>10$, suggesting a saturation threshold in the geometric amplification effect.

## 3. Results

### 3.1. The Coefficient of Thermal Expansion of Cylindrical Metamaterials

**Figure 6:**
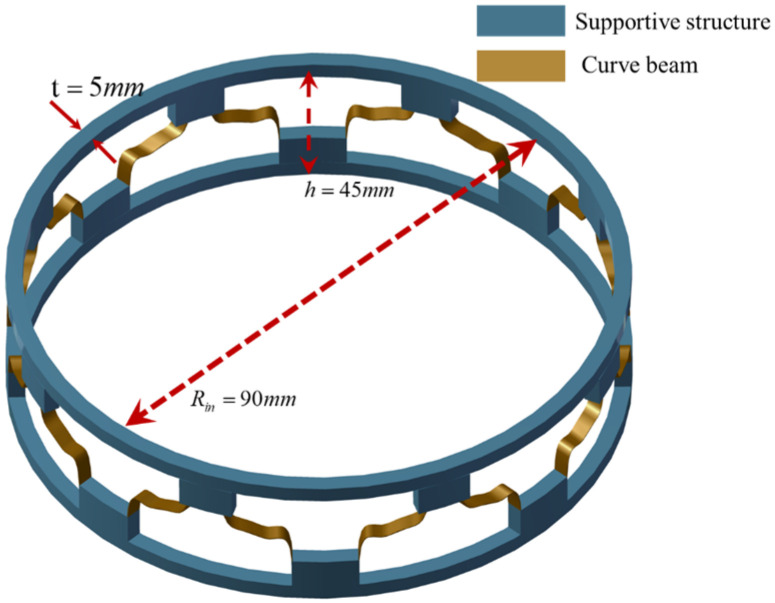
Parameter diagram of cylindrical metamaterials.

When the geometric parameters of the curved-beam unit cell is set to $l_{c}=20\mathrm{mm}$ and h=15mm, and elastic modulus is $E_{c}=E_{s}=6.6\mathrm{GPa}$, the structure exhibits ideal adjustable CTE characteristics. Leveraging this optimization, the curved-beam unit cell is assembled into a cylindrical metamaterial structure, with detailed structural parameters illustrated in Figure 6. Subsequently, a comprehensive analysis of the thermal expansion characteristics of the cylindrical metamaterial structure is conducted, focusing on the relationship between the thermal expansion coefficients of the support structure and the curved beam.

The mechanical performance of the cylindrical metamaterial was numerically investigated using the commercial finite element software ABAQUS 2024. The load–displacement curves were obtained through the static analysis module, while the vibration isolation performance was evaluated using the steady-state dynamic analysis module. To avoid excessively sharp resonance peaks, which would hinder a stable characterization of the vibration-transmission and isolation behavior, a uniform damping ratio of 0.02 was adopted in the analysis. The finite element model was established using C3D8R solid elements, with a total of 122,500 elements. In particular, the curved-beam region was discretized into three element layers through the wall thickness, with an element size of 0.1 mm for each layer. Regarding the boundary conditions, displacement constraints were applied in the cylindrical coordinate system for the cylindrical metamaterial structure. The bottom boundary was constrained with only the radial displacement released, while an equivalent concentrated load corresponding to a mass of 19.1 kg was applied at the top.

**Figure 7:**
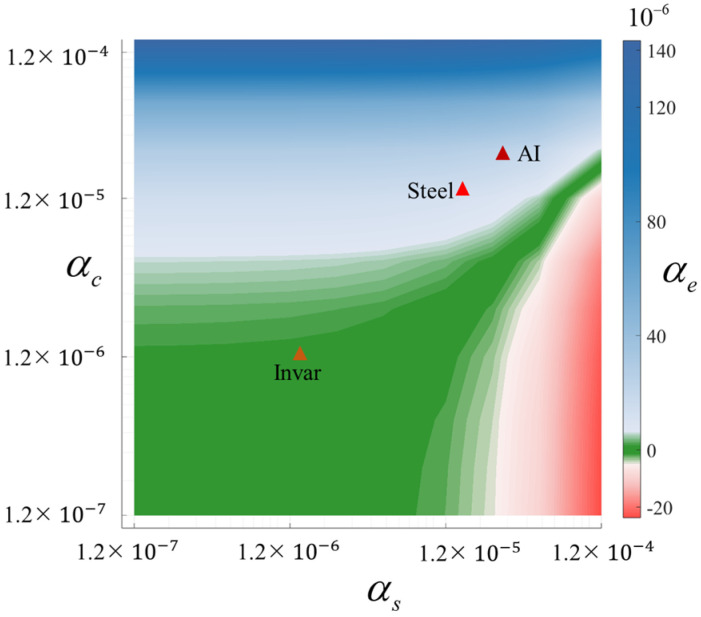
Influence of thermal expansion coefficients of the curved beam and support structure on the equivalent thermal expansion coefficient of cylindrical metamaterials.

Figure 7 clearly demonstrates that different combinations of thermal expansion coefficients significantly influence the equivalent thermal expansion behavior of cylindrical metamaterials. The study establishes $\pm 1\times 10^{-6}/{}^{\circ}C$ as the critical threshold for distinguishing different thermal expansion states. Specifically, when the thermal expansion coefficients of both the curved beam and supporting structure are below the critical value $1.2\times 10^{-6}/{}^{\circ}C$, the cylindrical metamaterial exhibits ZTE characteristics in the vertical direction, though this state requires specific material parameter combinations to achieve. Notably, even when thermal expansion coefficients exceed the threshold $1.2\times 10^{-6}/{}^{\circ}C$, the desired thermal expansion performance can still be attained through rational dual-material design. However, as the material thermal expansion coefficients continue to increase, the design space satisfying ZTE characteristics progressively narrows.

### 3.2. Thermal Stability Analysis of Cylindrical Metamaterials

While the achievement of the QZS and ZTE properties enables effective suppression of vertical displacement under thermal loading, the presence of temperature fields inevitably induces thermal stresses and displacements within the curved beams. These thermally induced effects may significantly compromise the QZS characteristics and stable vibration isolation performance of the structure. Consequently, a systematic investigation into the load-bearing capacity and vibration performance of cylindrical metamaterials with varying ZTE coefficient combinations becomes imperative for both theoretical understanding and practical applications.

**Figure 8:**
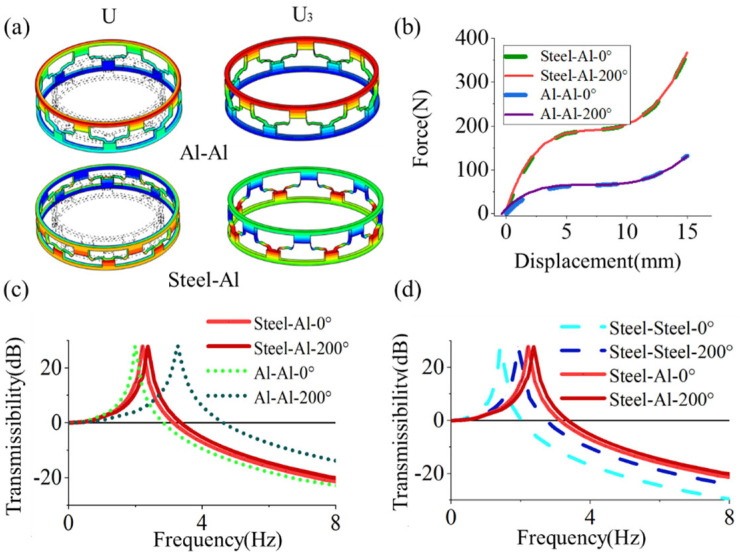
(**a**) Radial and axial deformation of cylindrical metamaterials composed of Al–Al and Al–Steel materials (Al–Al $\alpha_{s}=\;\alpha_{c}=2.3\times 10^{-5}\;{}^{\circ}C$ and Al–Steel $\alpha_{s}=2.3\times 10^{-5}\;{}^{\circ}C,\;\alpha_{c}=1.2\times 10^{-5}\;{}^{\circ}C$) at 200 °C. (**b**) Force–displacement characteristics of cylindrical metamaterials under thermal variations. (**c**) Resonance peak shift in Al–Al and Al-Steel cylindrical metamaterials under a temperature gradient. (**d**) Resonance peak shift in Steel–Steel and Al–Steel cylindrical metamaterials under a temperature gradient.

As shown in Figure 8a, the cylindrical metamaterial exhibits pronounced thermo-mechanical behavior distinct from that of a planar curved-beam unit. For the Al–Al ($\alpha_{s}=\alpha_{c}=2.3\times 10^{-5}/{}^{\circ}C$) cylindrical metamaterial, thermal loading induces significant radial and axial expansion. Under such conditions, the curved beams undergo torsional and tensile deformation, leading to an alteration of the load transfer path. As shown in Figure 8b, this thermally induced geometric deformation results in a change in overall structural stiffness.

In contrast, for the Al–Steel ($\alpha_{s}=2.3\times 10^{-5}/{}^{\circ}C,\;\alpha_{c}=1.2\times 10^{-5}/{}^{\circ}C$) cylindrical metamaterial, the mismatch in coefficients of thermal expansion between the two constituent materials plays a critical role. The deformation of the curved beams is constrained by the supporting structure due to differential thermal expansion. Consequently, as shown in Figure 8b, the beam deformation remains relatively small, and the variation in global structural stiffness is correspondingly limited.

In contrast, for the Al–Steel ($\alpha_{s}=2.3\times 10^{-5}/{}^{\circ}C,\;\alpha_{c}=1.2\times 10^{-5}/{}^{\circ}C$) cylindrical metamaterial, the mismatch in thermal expansion induces radial and axial stresses within the supporting structure under thermal loading, resulting in torsional and compressive deformation of the beams. The load–displacement curves in Figure 8b show only a small difference in curved-beam deformation between thermo-mechanical and room-temperature loading conditions. This indicates that overall stiffness is largely temperature-independent, as the main load-bearing upper beam undergoes only slight thermal deformation.

Figure 8c compares the vibration isolation performance of Al–Al and Al–Steel metamaterial. Under initial conditions (0 °C), their resonance peaks are at 1.99 Hz and 2.20 Hz, respectively. At 200 °C, the peaks shift to 3.27 Hz (64.32% shift, $\Delta f/f_{0\;{}^{\circ}C}$) and 2.37 Hz (7.72% shift). Similarly, Figure 8d compares Steel–Steel and Steel-Al metamaterial structure. The initial resonance of the Steel–Steel structure is 1.41 Hz. At 200 °C, its peak shifts to 1.96 Hz, representing a 39.01% frequency shift.

**Figure 9:**
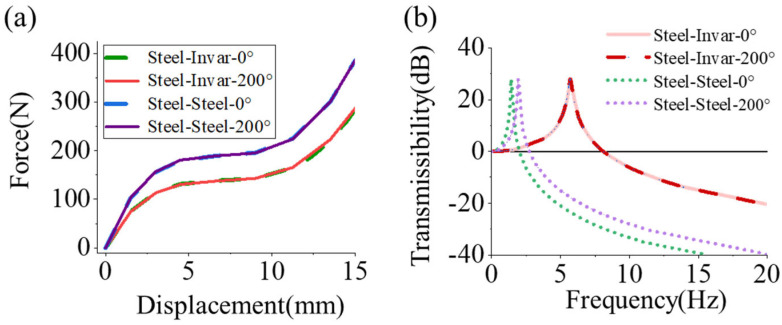
(**a**) Force–displacement curve of metamaterials (Steel–Steel $\alpha_{s}=\;\alpha_{c}=1.2\times 10^{-5}\;{}^{\circ}C$ and Steel–Invar $\alpha_{s}=1.2\times 10^{-5}\;{}^{\circ}C,\;\alpha_{c}=1.2\times 10^{-6}\;{}^{\circ}C$) under thermal variations. (**b**) Resonance peak shifts in cylindrical metamaterials subjected to temperature gradients.

Further investigations were conducted on cylindrical metamaterials utilizing a material combination with Steel–Invar ($\alpha_{s}=1.2\times 10^{-5}\;{}^{\circ}C,\;\alpha_{c}=1.2\times 10^{-6}\;{}^{\circ}C$). The results indicate that the deformation profile is similar to that shown in Figure 8a. Figure 9a presents the load–displacement curves for both the Steel–Steel and Steel–Invar metamaterials, revealing that temperature has a small effect on their stiffness. Furthermore, Figure 9b demonstrates that the Steel–Invar cylindrical metamaterial exhibits superior vibrational stability under thermal variations. Specifically, its center frequency shifts only slightly from 5.58 Hz at 0 °C to 5.61 Hz at 200 °C. Notably, due to the lower elastic modulus of the Invar alloy, the structure deviates from the QZS region under identical loading conditions. This deviation causes a larger shift in the center frequency compared to the Steel–Steel metamaterial at room temperature.

## 4. Conclusions

This study systematically investigates the effects of temperature on the load-bearing and vibration isolation characteristics of cylindrical metamaterials. To overcome the progressive degradation of vibration isolation performance caused by thermo-mechanical coupling, an innovative dual-zero metamaterial design was developed, successfully integrating QZS and ZTE properties. Utilizing the CBCM, the study accurately predicted the thermo-mechanical deformation behavior of spline curved beams, demonstrating that equivalent loads from supporting structures can effectively suppress vertical thermal displacement. By implementing a dual-material system (such as Al–Steel and Steel–Invar), the design leverages thermal expansion mismatch to constrain temperature-induced geometric distortions, thereby stabilizing the overall structural stiffness. Simulation results highlight the marked superiority of this approach: the traditional single-material (Al–Al) structure exhibits a significant resonance peak shift of up to 64.32% at 200 °C, while the zero-stiffness design of the dual-material (Al–Steel) has a resonance peak shift of only 7.72%. Notably, the Steel–Invar configuration exhibited superior vibrational stability, shifting its center frequency only slightly from 5.58 Hz to 5.61 Hz at 200 °C. The design is particularly promising for engineering systems requiring simultaneous load-bearing capacity, low-frequency vibration isolation, and thermal stability, such as precision instruments, aerospace payload platforms, and other thermally sensitive equipment. This design methodology enables the synergistic optimization of load-bearing capacity and low-frequency vibration isolation, providing a useful reference for the development of engineering metamaterials for extreme-temperature environments.

## Figures and Tables

**Table 1:** Comparison of the present work with representative previously published studies on metamaterials.

Ref.	Metastructure Form	Thermal Adjustment	Vibration Isolation	Main Gap Relative to the Present Work
Wei [26] (2021)	Cylindrical	Multiple materials	No discussion	No ZTE design and low-frequency vibration isolation function
Li [20] (2023)	Springs + curved beam	Metal phase control	<150 Hz	Mainly focuses on the tunability of the bandgap under temperature influence
Yu [27] (2023)	Star-shaped lattice	Multiple materials	>1000 Hz	High-frequency thermal stability analysis of cells, without low-frequency vibration isolation function
Cai [11] (2025)	Curved beam	No discussion	<100 Hz	Focuses on the control of bandgap characteristics, without addressing the vibration isolation stability under thermal conditions.
Zeng [21] (2025)	Curved beam	Invar alloy	<100 Hz	narrow range of material choices and difficulties in modulating the thermal expansion coefficient

## Data Availability

The original contributions presented in this study are included in the article. Further inquiries can be directed to the corresponding authors.
